# Gut Microbiota Dysbiosis in Preeclampsia: Mechanisms, Biomarkers, and Probiotic-Based Interventions

**DOI:** 10.1155/mi/3010379

**Published:** 2025-08-11

**Authors:** Yefang Zhao, Bingjie Wang, Xujing Wei, Dengxiang Liu, Ruiping Wang, Huashu Ma, Zongxu Qiao, Nana Kong, Jinhui Feng, Dan Cui, Shaoke Hou, Hongzhen Zhang

**Affiliations:** ^1^Department of Obstetrics, Xingtai People's Hospital, Affiliated Hospital of Hebei Medical University, Xingtai, Hebei, China; ^2^Department of Proctology, Xingtai People's Hospital, Affiliated Hospital of Hebei Medical University, Xingtai, Hebei, China; ^3^Department of Obstetrics and Gynecology, First Hospital of Hebei Medical University, Shijiazhuang, Hebei, China; ^4^Cancer Laboratory, Xingtai People's Hospital, Affiliated Hospital of Hebei Medical University, Xingtai, Hebei, China

**Keywords:** fecal microbiota transplantation, gut microbiota, inflammatory factor, intestinal barrier, preeclampsia

## Abstract

**Background:** This study aimed to investigate the impact of fecal microbiota transplantation (FMT) on gut microbiota composition and serum inflammatory factors in a murine model.

**Methods:** Female C57BL/6J mice (*n* = 60) were divided into four groups: control (Con), negative (Neg), normal transplantation (NT), and preeclampsia transplantation (PET). The Con group received no treatment, while the Neg, NT, and PET groups were administered a triple antibiotic regimen (ampicillin, neomycin sulfate, and metronidazole) for 14 days to deplete gut microbiota. Following antibiotic treatment, FMT was performed: the NT group received fecal microbiota from healthy pregnant women and the PET group received microbiota from severe preeclampsia patients. Fecal samples and serum were collected for 16S rRNA sequencing and inflammatory factor analysis, respectively.

**Results:** Significant differences in gut microbial composition were observed between the PET group and other groups, with enriched taxa such as *Coprococcus*, *Bacillales*, and *Staphylococcus* in the PET group. Conversely, taxa such as *Helicobacter* and *Klebsiella* were more abundant in the fecal microbiota of mice in the NT group. Furthermore, serum levels of lipopolysaccharide (LPS), tumor necrosis factor-alpha (TNF-α), and interleukin-6 (IL-6) were markedly elevated in the PET group compared to the control, negative, and NT groups. Transplantation with fecal bacteria from preeclampsia patients leads to significant alterations in gut microbiota composition and increased serum inflammatory factors levels in mice.

**Conclusion:** These findings provide insights into the relationship between gut microbiota and inflammatory processes in preeclampsia and underscore the potential therapeutic implications of FMT in modulating gut microbiota dysbiosis and inflammatory responses.

## 1. Introduction

The gut microbiota refers to the complex and vast microbial community residing in the human digestive tract, comprising approximately 1 × 10^13^ microbial species, including over 250 types of bacteria, viruses, fungi, and archaea [1]. Gut microbiota and the host maintain a highly mutualistic and dynamically balanced relationship throughout human life [2]. It is intricately associated with human development, nutrient absorption, immunity, and diseases [3]. Gut microbiota dysbiosis occurs when there is a significant shift in bacterial proportions or excessive growth of certain bacteria [4]. Gut microbiota dysbiosis is often characterized by reduced microbial richness and diversity, although this is not universally observed. Additionally, it can involve an overgrowth of bacteria that produce lipopolysaccharide (LPS), including but not limited to Proteobacteria [5]. The excessive activation of Toll-like receptors (TLRs) by invasive bacteria leads to the overexpression of inflammatory cytokines, resulting in epithelial injury and chronic inflammation [6]. Previous studies have confirmed that gut microbiota played an important role in the occurrence and development of metabolic diseases such as hypertension and diabetes [7]. The fecal bacteria of essential hypertensive rats showed a significant reduction in the abundance and diversity of bacterial flora, including an increase in the proportion of Firmicutes and Bacteroidetes and a decrease in butyrate-producing bacteria. After the application of minocycline, it was found that the proportion of Firmicutes and Bacteroidetes was reduced, the intestinal flora was rebalanced, and blood pressure was also reduced [8].

Our previous study [9] demonstrated significant differences in gut microbiota composition and serum inflammatory factor levels between normal pregnant women and preeclampsia patients. However, it established correlation rather than causation. Fecal microbiota transplantation (FMT) involves the transfer of fecal bacterial suspension from a donor into the recipient's gut, directly altering the recipient's gut bacterial composition and potentially establishing a link between the altered microbiome and diseases [10, 11]. Previous research has shown that transferring the gut microbiota from healthy individuals and hypertensive patients into sterile mice resulted in increased blood pressure in the hypertensive group [12]. Another study transplanted stool samples from healthy pregnant women with gestational diabetes and normal blood glucose levels into germ-free mice, demonstrating distinct colonization patterns of gut microbiota and increased blood glucose levels in germ-free mice receiving fecal bacteria transplantation from gestational diabetes mellitus patients [13]. Both studies confirmed the causal relationship between gut microbiota and disease.

Given that C57BL/6J pseudo-aseptic mice are easily obtainable, easy to feed, cost-effective, and yield consistent results compared to sterile mice [14], this study employed triple antibiotic therapy to deplete the intestinal flora of C57BL/6J mice. Subsequently, the intestinal flora from both preeclampsia patients and healthy pregnant women was transplanted into these pseudo-aseptic mice. After transplantation, the intestinal flora and serum inflammatory factor levels in mice were assessed to elucidate the relationship between intestinal flora and inflammatory factors in preeclampsia patients, thereby providing a theoretical basis for understanding the pathogenesis and treatment of preeclampsia.

## 2. Materials and Methods

### 2.1. Donor Clinical Data

Three severe preeclampsia patients and three healthy pregnant women were randomly selected as fecal bacteria donors (Table A1). Donors were screened for any underlying health conditions or medication use that could affect their gut microbiota. The protocol was approved by The Ethics Committee of Xingtai People's Hospital (approval number: 2023[018]) and the study was performed in accordance with the Helsinki II Declaration.

### 2.2. Animals

Female C57BL/6J mice (*n* = 60), aged 6 weeks with a body weight of 16–20 g, were obtained from Charles River, Inc., Beijing, China. Mice were acclimatized for 1 week prior to the start of the experiment to ensure adaptation to laboratory conditions. The protocol was approved by Xingtai People's Hospital Animal Care and Use Committee (Permission number: 2023[018]). All experiments adhered to the ARRIVE guidelines for reporting animal research.

### 2.3. Euthanasia

At the conclusion of the experiment, mice were deeply anesthetized via intraperitoneal injection of pentobarbital sodium (100 mg/kg body weight). Euthanasia was confirmed by cervical dislocation, following institutional ethical standards. This method ensured minimal distress and complied with the AVMA Guidelines for the Euthanasia of Animals.

### 2.4. Preparation of Fecal Bacterial Solution

Clinical fecal sample collection was performed in a GMP-certified clean laboratory. The feces of three patients with severe preeclampsia were mixed to prepare fecal bacterial solution and the fecal bacterial solution of normal pregnant women was prepared in the same way. Fresh feces from the selected donors were collected into sterile 50 ml centrifuge tubes and mixed with sterile PBS solution (1 g of fresh feces dissolved in 5 ml of sterile PBS). The mixture was vortexed, centrifuged at 1000 × *g* for 2 min, and the supernatant was collected and stored in 1.5 ml centrifuge tubes at −80°C for future use.

### 2.5. FMT

FMT experiment in mice was conducted under SPF conditions. C57BL/6J mice were divided into four groups: control (Con), negative (Neg), normal transplantation (NT), and preeclampsia transplantation (PET) groups. The Con group had ad libitum access to food and water without medical intervention. The Neg, NT, and PET groups received antibiotics (ampicillin 1 g/L, neomycin sulfate 1 g/L, and metronidazole 1 g/L) in drinking water for 14 consecutive days. Subsequently, FMT was performed, with fecal bacterial solution from the selected donors administered orally to the mice. The gavage volume was based on 0.1 ml/10 g. After continuous gavage for 3 days, it was changed to twice a week for a duration of 6 weeks. Fresh fecal samples were collected on day 43 posttransplantation and stored at −80°C until use.

### 2.6. Sample Collection and 16S rRNA Sequencing

Fecal samples were collected into sterile tubes and stored at −20 to −40°C before transfer to the laboratory on dry ice. DNA extraction was performed using the OMEGA Soil DNA Kit and 16S rRNA gene amplicon sequencing was carried out using PCR amplification of the V3–V4 region. Forward primer was 338F (5′-ACTCCTACGGGAGGCAGCA-3′) and the reverse primer was 806R (5′-GGACTACHVGGGTWTCTAAT-3′). Bioinformatics analysis was performed using QIIME2 (https://docs.qiime2.org/2019.4/tutorials/) and R packages (v3.2.0).

### 2.7. Quantification of Serum Inflammatory Factors

Serum concentrations of interleukin-6 (IL-6), tumor necrosis factor-alpha (TNF-α), and LPS were measured using enzyme-linked immunosorbent assay (ELISA) kits from Abcam and Proteintech. In the 7th week of the experiment, the urine of the mice in the four groups was collected and the urine protein was determined. Measurement of serum inflammatory factors: The samples were measured three times in duplicate and the average value was taken. The detection limit of LPS was 0.313-20 μg/ml and the detection limit of IL-6 and TNF-α was 15.6–1000 pg/ml. The LPS ELISA kit was purchased from Wuhan Fine Biotech Co., Ltd, and the IL-6 and TNF-α ELISA kits were purchased from Leader in Biomolecular Solutions for Life Science. LPS determination conditions: The extracted blood samples were placed in anticoagulation tubes, centrifuged at 4°C and 3500 × *g* for 15 min to obtain the supernatant. The sample information was labeled and immediately stored in a −80°C refrigerator for later use.

### 2.8. Statistical Analysis

The data were analyzed using SPSS and GraphPad Prism software. Normal distribution data were presented as mean ± standard deviation, while skewed distribution data were presented as median (25th–75th quartiles). Statistical significance was set at *p*  < 0.05. Detailed statistical methods including Wilcoxon's rank-sum test, Student's *t*-test, one-way analysis of variance, Pearson's *χ*^2^ test, Fisher's exact test, and ANOSIM were employed to evaluate differences among groups.

## 3. Results

### 3.1. Comparison of Gut Microbiota Levels Among Four Groups of Mice

**Figure 1:**
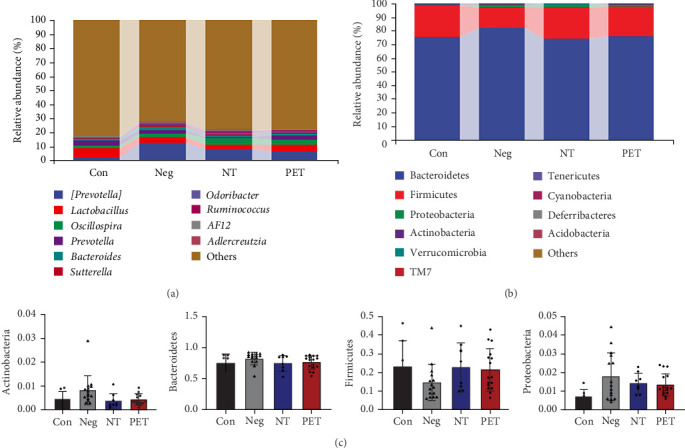
Comparison of gut microbiota levels among four groups of mice. (A) The distribution of top 10 bacteria at the genus level in the four groups. (B) The distribution of top 10 bacteria at the phylum level in the four groups. (C) The top four bacteria at the phylum level in the four groups.

At the genus level, the predominant bacteria were *[Prevotella]*, *Lactobacillus*, *Oscillospira*, *Prevotella*, *Bacteroides*, *Sutterella*, *Odoribacter*, *Ruminnococcus*, *AF12*, *and Adlercreutzia* (Figure 1A). At the phylum level, the dominant bacteria were Bacteroidetes, Firmicutes, Proteobacteria, Actinobacteria, Verrucomicrobia, TM7, Tenericutes, Cyanobacteria, Deferribacteres, and Acidobacteria (Figure 1B). There was no statistically significant difference in the abundance of bacteria at the top four bacteria at the phylum level in the four groups (*p*  > 0.05, Figure 1C).

### 3.2. Comparison of Gut Microbiota Diversity Among Four Groups

**Figure 2:**
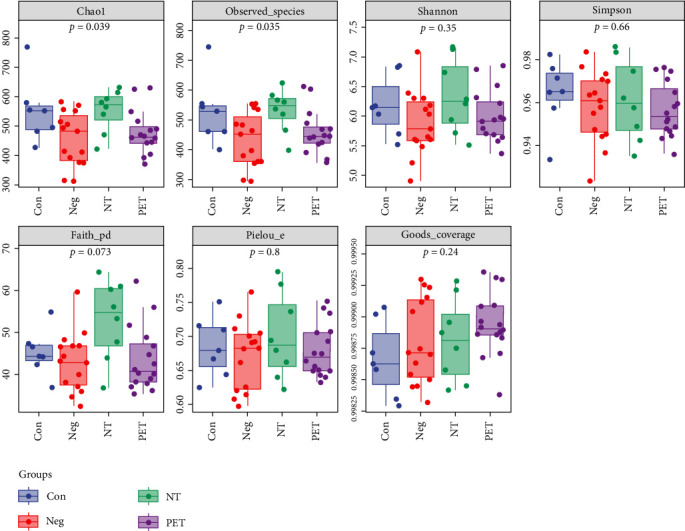
Varying alpha diversity of gut microbiota in the four groups.

Slight decreases in microbial alpha diversity assessed by the Chao 1 diversity index (*p*=0.039) and observed species diversity index (*p*=0.035) were observed in the PET group compared to the Con, Neg, and NT groups. However, no statistically significant differences were observed in the Shannon diversity index (*p*=0.35), Simpson diversity index (*p*=0.66), Faith's PD (*p*=0.073), Good's coverage (*p*=0.24), and Pielou e among the four groups (*p*=0.8, Figure 2).

**Figure 3:**
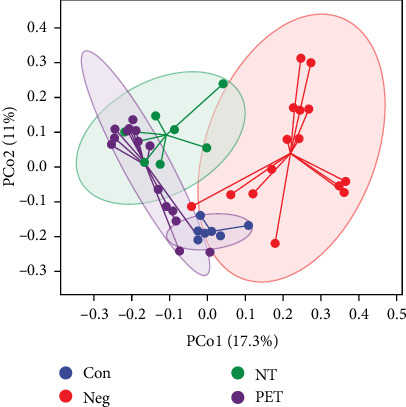
PCoA plot showing the dispersal of microbiota in beta diversity in the four groups.

Microbiota beta diversity was analyzed using Bray–Curtis distance analysis and principal coordinate analysis. The results revealed distinct separation among the four groups of samples (*R*^2^ = 0.307, *p*=0.001), with PC1 and PC2 contributing 17.3% and 11% to the variation, respectively (Figure 3).

### 3.3. Comparison of Differential Bacteria in Four Groups

**Figure 4:**
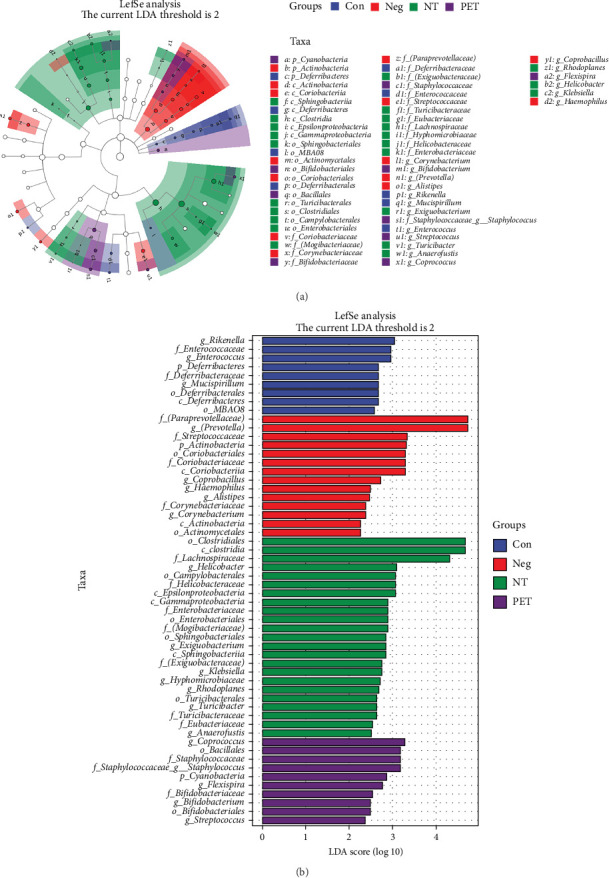
Differential bacteria comparison in four groups. (A) Four groups of taxonomic biomarkers. (B) The histogram of taxonomic biomarkers in the four groups.

To further investigate whether the gut microbiota of the PET group differed from the other groups, we applied LEfSe analysis to explore the variations and relative richness of the gut microbiota. The predominant bacteria in the fecal microbiota of PET mice were mainly concentrated in *Coprococcus*, *Bacillales*, *Staphylococcus*, *Cyanobacteria*, *Flexispira*, *Bifidobacterium*, and *Streptococcus*. In contrast, the NT group exhibited dominance in *Helicobacter*, *Klebsiella*, *Rhodoplanes*, *Exiguobacterium*, *Turicibacter*, and *Anaerofustis*. The Neg group displayed dominance in *ParaPrevotellaceae*, *Prevotella*, *Streptococcaceae*, *Actinobacteria*, *Coriobacteriales*, *Coriobacteriaceae*, *Coriobacteriia*, *Coprobacillus*, *Haemophilus*, *Alistipes*, *Corynebacterium*, *Actinobacteria*, and *Actinomycetales*. The Con group predominantly harbored *Rikenella*, *Enterococcus*, *Enterococcaceae*, *Deferribacteres*, *Mucispirillum*, *Deferribacterales*, *Defferibacteres*, and MBA08 (Figure 4). It can be concluded that the composition and structure of the gut microbiota differed among the four groups after receiving different fecal bacterial solutions.

### 3.4. Comparison of Serum LPS and Inflammatory Cytokine Levels in Con, Neg, NT, and PET Groups of Mice

The serum LPS concentrations in the Con, Neg, NT, and PET groups were 1.90 ± 0.93, 4.34 ± 1.63, 4.75 ± 2.61, and 10.26 ± 3.91 µg/ml, respectively, indicating highest level of serum LPS in the PET group (*p*  < 0.001). However, there was no statistically significant among the other three groups (*p*  > 0.05).

The serum TNF-α levels in the Con, Neg, NT, and PET groups were 9.99 ± 2.47, 10.96 ± 1.05, 9.88 ± 2.54, and 13.34 ± 1.07 pg/ml, respectively. The PET group exhibited significantly higher levels compared to the other three groups, with a statistically significant difference (*p*  < 0.001). In contrast, there was no statistically significant difference among the other three groups (*p*  > 0.05).

**Figure 5:**
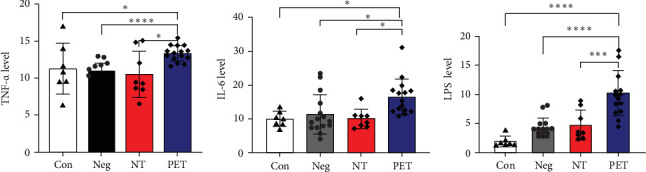
Serum Levels of LPS, TNF-α, and IL-6 in Con, Neg, NT, and PET groups. *⁣*^*∗*^*p* < 0.05, *⁣*^*∗∗∗*^*p* < 0.01, and *⁣*^*∗∗∗∗*^*p* < 0.001.

The serum IL-6 concentrations in the four groups were 10.04 ± 2.25, 11.38 ± 5.82, 10.07 ± 2.84, and 16.48 ± 5.33 pg/ml, respectively. The PET group showed significantly higher levels compared to the other three groups, with a statistically significant difference (*p*=0.005). However, there was no statistically significant difference among the other three groups (*p*  > 0.05, Figure 5).

**Figure 6:**
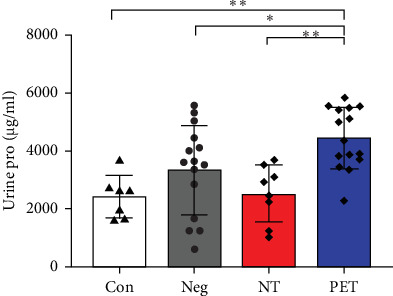
Quantitative comparison of urinary protein. *⁣*^*∗*^*p* < 0.05 and *⁣*^*∗∗*^*p* < 0.01.

The average urine protein contents in the Con group, Neg group, NT group, and PET group were 2421.75 ± 737.46, 3049.8.80 ± 1549.63, 2520.98 ± 987.31, and 4456.24 ± 1509.05 µg/ml, respectively. The average urine protein contents in the PET group was significantly higher than those of the other three groups (*p*  < 0.001), while there was no statistically significant difference among the other three groups (*p*  > 0.05, Figure 6).

## 4. Discussion

This study aims to elucidate the pathogenesis of preeclampsia from the perspective of gut microbiota. Previous studies have highlighted significant differences in the composition of gut microbiota and serum inflammatory factors between patients with severe preeclampsia and healthy pregnancies, indicating a correlation between different bacteria, functional pathways in gut microbiota, and serum inflammatory factors. But the causal relationship has not yet been confirmed. FMT experiments can confirm the causal relationship between gut microbiota and serum inflammatory factors. Therefore, we measured the intestinal flora and serum inflammatory factor levels of mice after FMT from patients with severe preeclampsia to demonstrate that the imbalance of intestinal flora can lead to an increase in serum inflammatory factor levels. The results revealed that the SPF mice given preeclamptic FMT exhibited a different composition of intestinal flora and increased serum levels of TNF-α, IL-6, LPS, and urine protein.

Sequencing of gut microbiota after FMT in four groups of mice revealed enrichment of Coprococcus, Staphylococcus, Bifidobacterium, and Streptococcus in the PET group. Staphylococcus, Streptococcus and Coprococcus were all gram-positive cocci, which can cause purulent infections and food poisoning in humans. Our previous research results also suggested that the enrichment of Streptococcus in the gut microbiota of preeclampsia patients was consistent with this result. The possible mechanism for the onset of preeclampsia is the overgrowth of harmful bacteria in the gut microbiota, which leads to damage to the integrity of intestinal epithelial cells and intestinal leakage. Increased secretion of LPS and release of inflammatory factors lead to the occurrence of preeclampsia. However, Jordan et al.'s [15] research results showed that the decrease in the abundance of Streptococcus in preeclampsia patients may increase the risk of preeclampsia in pregnant women, which was inconsistent with our research results. This may be due to the small number of severe preeclampsia patients selected and racial differences. While several studies have shown that the lack of Lactobacillus in maternal gut microbiome is closely related to the development of clinical features of preeclampsia, and our study also have shown that mice transplanted with preeclamptic fecal microbiota suspension show a reduced abundance of Lactobacillus [16]. Sun et al. [17] found that at 3 days and 1 week after oral probiotics, serum endotoxin level was significantly lower and higher levels of Lactobacillus in intestinal flora, which was consistent with our research. Lactobacillus is an anti-inflammatory species which is essential for producing short-chain fatty acids (SCFAs) such as butyrate, propionate, and acetate [18], which are crucial in maintaining immune homeostasis and gut barrier integrity. SCFAs mediate their effects through multiple molecular pathways. They act by binding to G-protein-coupled receptors (GPR41 and GPR43) on intestinal epithelial cells and immune cells, activating anti-inflammatory signaling pathways that suppress the nuclear factor kappa B (NF-κB) pathway [19].

After FMT in mice treated with antibiotics, we found that the levels of serum LPS, IL-6, and TNF-α in the group of mice transplanted with fecal microbiota fluid from patients with preeclampsia were higher than those in the other three groups. The possible mechanism is that intestinal microbiota imbalance causes downregulation of the expression of intestinal tight junction ZO-1 and Occludin genes and increased intestinal permeability leads to dislocation and movement of LPS at the intestinal barrier junction, entering the circulation and resulting in an increase in serum LPS levels [20]. Excessive LPS increase activates the inflammatory response through TLR 4 or defective enzyme cascade [21], thereby increasing the levels of serum pro-inflammatory factors.

LPS, also known as endotoxin, is a component of the outer membrane of gram-negative bacteria. Under normal circumstances, the intestinal barrier minimizes the passage of LPS from the gut into the systemic circulation [22]. Increased permeability of the intestinal barrier may lead to the dislocation and movement of LPS at the junction of the intestinal barrier, resulting in increased serum LPS levels [23]. Elevated LPS levels have been observed in obesity and other metabolic diseases, as well as in adipose tissue inflammation and pancreatic β-cell dysfunction [24–26]. Elevated LPS levels are considered one of the key links between gut microbiota and inflammation in metabolic syndrome [27–29]. In comparison with the other three groups, SPF mice transplanted with preeclamptic fecal microbiota suspension had higher serum LPS levels. The imbalance of intestinal flora may cause the downregulation of gut epithelium tight junctional gene expression, leading to increased intestinal permeability and elevated serum LPS levels [30]. Excessive LPS increase activates the inflammatory response through TLR 4 or defective proteasome cascades [31], resulting in increased levels of serum pro-inflammatory factors.

Our study also found that the SPF mice given preeclamptic FMT exhibited an increased urine protein than other groups, which is consistent with the clinical symptom of patients with preeclampsia and the fecal bacterial solution of three severe preeclampsia patients also had a high 24 h urinary protein quantity. This study demonstrated that after FMT, the PET group exhibited a different gut microbiota composition and increased levels of endotoxin and inflammatory factors. Previous studies have revealed differences in the composition of gut microbiota between preeclampsia and normotensive pregnant women, suggesting independent risk factors for the development of placental abnormalities and hypertensive disorders of pregnancy [32]. Several studies have shown that the lack of Lactobacillus in maternal gut microbiome is closely related to the development of clinical features of preeclampsia and mice transplanted with preeclamptic fecal microbiota suspension show a reduced abundance of Lactobacillus [16]. Currently, oral probiotics to prevent hypertensive disorders complicating pregnancy are still a “hot topic” [33]. One study showed that probiotics LC40 and *Bifidobacterium* breve CECT7263 (BFM) prevented microecologicaldysbiosis, endothelial dysfunction, and the development of hypertension in patients with hereditary hypertension [34]. Another animal experiment [17] demonstrated that probiotics could effectively reduce the level of serum endotoxin in preeclampsia rats, improving the body's ability to clear metabolites and the colonization ability of intestinal flora, maintaining the stability of intestinal flora, enhancing vascular endothelial function, and reducing blood pressure and inflammatory response.

Therefore, our study provides a theoretical basis for the supplementation of probiotics to improve gut microbiota imbalance. Probiotic supplementation in pregnant women or high-risk groups may reduce or alleviate the onset of preeclampsia. FMT is also expected to be a means to treat or prevent the onset of preeclampsia, but it is still in the experimental stage. However, there are differences in the regulation of intestinal flora in pregnant women by different probiotic strains [35]. Further extensive and in-depth research is needed to identify probiotic strains with the ability to improve the onset of preeclampsia.

In conclusion, this study offers valuable insights into the supplementation of probiotics to improve gut microbiota disorders. Beneficial bacteria of the intestine can be formulated into pills or capsules. Probiotic supplementation in pregnant women or high-risk groups may reduce or alleviate the onset of preeclampsia. Fecal bacteria transplantation is also anticipated to be an effective means to treat or prevent the onset of preeclampsia, although it remains in the experimental stage. However, different probiotic strains may differ in their regulation of intestinal flora in pregnant women, necessitating further extensive and in-depth studies on how to screen out probiotic strains that can improve the onset of preeclampsia.

Currently, there are relatively few researches on its causal relationship and there are also few studies exploring whether there are differences in gut microbiota when fecal microbiota from preeclampsia patients is transplanted into mice. In our study, we divided mice into four groups for research, highlighting that mice transplanted with fecal microbiota from preeclampsia patients exhibit different gut microbiota and elevated levels of serum inflammatory factors. This provides a theoretical basis for the onset and prevention of preeclampsia.

It is essential to acknowledge the limitations of our study. First, although the fecal bacteria transplantation experiment confirmed that the imbalance of gut microbiota can increase serum inflammatory factors, no causal verification experiment was conducted. Second, the intestinal barrier function of mice after fecal bacteria transplantation was not measured in this study. Last, complete feces were used in the fecal bacteria transplantation experiment and the specific components causing the onset of preeclampsia have not yet been determined. Further studies can explore the correlation between specific components of fecal bacteria and the onset of preeclampsia.

## 5. Conclusion

This study demonstrates that FMT from preeclampsia patients alters the gut microbiota composition and increases serum inflammatory factors in mice. The findings suggest that gut microbiota imbalance may contribute to the pathogenesis of preeclampsia. Probiotic supplementation and FMT show potential as therapeutic strategies to prevent or treat preeclampsia, warranting further investigation.

## Figures and Tables

**Table 1:** Clinical characteristics of fecal microbiota transplantation donors.

Item	N1	N2	N3	PE1	PE2	PE3
Age (years)	34	37	35	31	33	30
Gravidity	3	3	3	4	3	3
Parity	1	1	1	2	2	0
Gestational weeks on delivery (weeks)	38^+4^	38^+6^	38^+5^	34^+3^	35	34
BMI (kg/m^2^)	22.9	30.1	26.8	31.1	32.0	26.6
Birth weight	2710	3640	3270	1430	1880	1540
Systolic BP (mmHg)	95	119	96	150	170	145
Diastolic BP (mmHg)	65	85	64	110	105	95
Urine protein (—4+)	—	—	—	3+	3+	3+
Urine protein content (g/L)	—	—	—	3.85	4.767	0.999
24 h urine protein content	—	—	—	7.315	10.24	3.546
Edema (—4+)	—	—	—	2+	3+	3+
WBC (10^9^/L)	8.73	6.58	8.38	15.22	11.12	9.90
Hb (g/L)	120	134	119	136	109	119
PLT (10^9^/L)	195	139	201	319	262	197
PT	9.9	9.8	10.5	8.8	9.3	10.0
APTT	32.1	30.2	29.3	27.3	27.2	27.7
Fib (g/L)	4.62	4.63	4.12	2.79	3.54	3.82
D-dimer (μg/ml)	2.11	1.19	0.98	1.04	0.64	0.69
ALT (U/L)	11	12.2	9.8	39.7	11.7	12.3
AST (U/L)	18.5	19.7	14.2	27.6	19.0	21.3
TBA (μmol/L)	0.8	1.5	4.0	2.5	2.9	3.8
TP (g/L)	69	67.5	62.7	53.9	61.4	69.0
ALB-s (g/L)	35.6	34.2	36.4	29.2	33.3	35.8
ALP (U/L)	103.1	104.6	80.9	82.8	78.5	118.0
TBIL (μmol/L)	7.9	7.2	8.4	6.2	3.0	3.3
GLU (mmol/L)	3.68	4.10	4.27	3.27	4.09	3.89
UA (μmol/L)	350	389	375	488	425	444
LDH (U/L)	183.2	186.5	185.6	268.9	198.0	151.2
CREA (μmol/L)	55.8	62.9	49.8	53.2	54.3	58.5
UREA (mmol/L)	4.74	3.53	3.36	5.11	5.93	3.99

*Note*: The format “w^+d^” represents weeks and days of gestation.

Abbreviations: APTT, activated partial thromboplastin time; PT, prothrombin time.

## Data Availability

The data that support the findings of this study are within the article. Further data are available from the corresponding author upon reasonable request.
